# A20 DIETARY ANTIGENS DELIVERED TO MICE IN EARLY LIFE INDUCE ANTIGEN-SPECIFIC REGULATORY AND TH1 CELLS AND THE DEVELOPMENT OF ORAL TOLERANCE

**DOI:** 10.1093/jcag/gwae059.020

**Published:** 2025-02-10

**Authors:** R D FitzPatrick, A M Przydatek, D M Gatti, R A Cartwright, N J Norton, J M Lane, L A Reynolds

**Affiliations:** University of Victoria, Victoria, BC, Canada; University of Victoria, Victoria, BC, Canada; University of Victoria, Victoria, BC, Canada; University of Victoria, Victoria, BC, Canada; Biochemistry & Microbiology, University of Victoria, Victoria, BC, Canada; University of Victoria, Victoria, BC, Canada; University of Victoria, Victoria, BC, Canada

## Abstract

**Background:**

Oral tolerance is the active suppression of immune responses to antigens that are first encountered in the gut. When this process fails, food allergies or celiac disease can arise. Oral tolerance develops largely in early life, but despite this it has historically been studied using *adult* rodent models.

**Aims:**

Our research aims to develop a robust mouse model to elucidate mechanisms of oral tolerance development in *early life*.

**Methods:**

Mice were orally gavaged with ovalbumin (OVA) as a model food antigen or water as a control in an early life, pre-weaning window (2-3 weeks old). Following systemic challenges with OVA, oral tolerance was assessed by measuring circulating antibody levels (ELISAs) and cytokine responses of *ex vivo* stimulated splenocytes and gut-draining mesenteric lymph node (MLN) cells (cytometric bead arrays). To characterize OVA–specific T helper cell responses, OT-II cells (OVA-specific CD4^+^ T cells) were adoptively transferred into mouse pups and their phenotypes were assessed in the MLNs and spleen by flow cytometry following oral gavages of OVA or control water.

**Results:**

BALB/cJ and C57BL/6J mice orally gavaged with OVA in early life had reduced levels of OVA-specific antibodies compared to controls after systemic challenges with OVA, indicating that oral tolerance had developed. Further, we determined the minimum dosage of oral OVA required to confer oral tolerance during early life. We found a suppression of cytokine production in response to OVA-stimulation in cultures of MLN cells or splenocytes, from mice orally exposed to OVA (‘tolerized’) in early life compared to cell cultures from control mice.

We found an increased frequency of OVA-specific regulatory T cells in the MLNs and spleen in response to oral OVA exposure compared to mice exposed to control water. We also found an increase in the frequency of OVA-specific Th1 cells along with an increase in OVA-specific CD4^+^ T cells that are producing IFNγ when pups were exposed to oral OVA compared to control water.

**Conclusions:**

We have developed a robust early life mouse model of oral tolerance. Our data suggests that oral tolerance is induced during early life in part following the central dogma of oral tolerance development elucidated in adult mice: with the induction of food-antigen specific regulatory T cells. However, we have also identified an increase in the frequency of food antigen-specific Th1 cells in the MLNs and spleen in response to oral antigen exposure during an early life, pre-weaning window. Future work will determine if IFNγ-expressing Th1 cells are essential for the development or maintenance of oral tolerance.

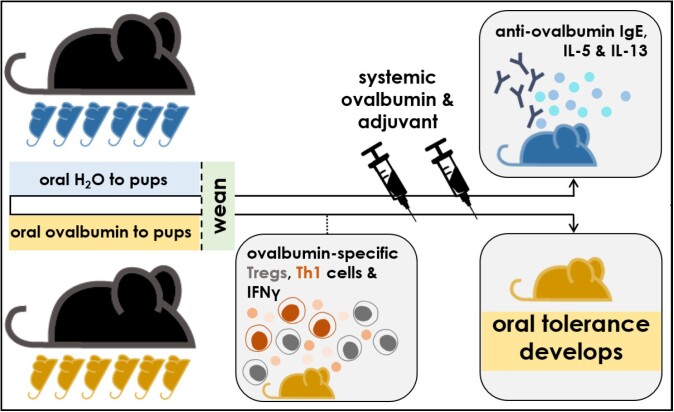

Oral tolerance development in early life.

**Funding Agencies:**

CIHRTRIANGLE

